# Difference in autonomic nervous effect of blue light depending on the angle of incidence on the eye

**DOI:** 10.1186/s13104-020-04988-5

**Published:** 2020-03-10

**Authors:** Emi Yuda, Yutaka Yoshida, Norihiro Ueda, Junichiro Hayano

**Affiliations:** 1 Tohoku University Graduate School of Engineering, Aoba 6-6-05 Aramaki Aoba-ku, Sendai, 980-8759 Japan; 2 Nagoya City University Graduate School of Design and Architecture, Kita Chikusa 2-1-10 Chikusa-ku, Nagoya, 464-0083 Japan; 3 Department of Medical Education, Nagoya City University Graduate School of Medical Sciences, 1 Kawasumi Mizuho-cho Mizuho-ku, Nagoya, 467-8601 Japan

**Keywords:** Autonomic nervous system, Blue light, Intrinsically-photosensitive retinal ganglion cell, Light-emitting diode, Non-image forming function, Smartphone, Solid-state lighting

## Abstract

**Objective:**

Blue light has been attributed to the adverse biological effects caused by the use of smartphones and tablet devices at night. However, it is not realistic to immediately avoid nighttime exposure to blue light in the lifestyle of modern society, so other effective methods should be investigated. Earlier studies reported that inferior retinal light exposure causes greater melatonin suppression than superior retinal exposure. We examined whether the autonomic responses to blue light depends on the angle of incidence to the eye.

**Results:**

In eight healthy subjects, blue light from organic electroluminescent lighting device (15.4 lx at subjects’ eye) was exposed from 6 angles (0º, 30º, 45º, 135º, 150º, and 180º) for 5 min each with a 10-min interval of darkness. After adjusting the order effect of angles, however, no significant difference in heart rate or autonomic indices of heart rate variability with the angle of incidence was detected in this study.

## Introduction

While light is an image forming signal for vision, light reaching the retina also causes various non-image forming reactions, such as pupil size adjustment, increased vigilance, autonomic nervous system arousal, and circadian clock adaptation [1]. These non-image forming functions were known to be mediated by intrinsically photosensitive retinal ganglion cells (ipRGCs) [2, 3], which express melanopsin as the photopigment. Since melanopsin has a specific sensitivity to blue wavelength light around 480 nm [4], blue light stimulation to the ipRGCs is recognized as a central mediator of the non-image-forming response to light [5–7].

In recent years, blue light has also been attributed to the adverse biological effects caused by the use of smartphones and tablet devices at night [8–11]. Accumulating evidence shows that the use of these light-emitting devices immediately before bedtime has negative impact on sleep [12, 13]. The displays of these devises use solid-state lighting (SSL), most of which emits much blue light even though they appear to emit white light [10, 11].

SSLs, however, have many benefits over traditional light sources and they deeply penetrate into the lifestyle, education, and industry of modern societies that are operating 24 h of a day. It seems impractical to immediately avoid exposure to blue light at night. We therefore need to investigate and accumulate our knowledge to control the negative biological effects of blue light. Previous studies have suggested the influences of the distance to light source emitting blue light [14] and the angle of incidence on the eye. Studies reported that white light exposure to the inferior retina causes greater melatonin suppression than exposure to the superior retina [15, 16]. In this study, we examined whether the autonomic effects of blue light depend on the incidence angle on the eye.

## Main text

### Methods

The present study was performed according to the protocol approved by the Ethics Review Committee of Nagoya City University Graduate School of Medical Sciences and Nagoya City University Hospital (approved No. 60160164).

#### Subjects

We studied eight subjects (mean age ± SD, 22 ± 3 year, seven males and a female) who had normal color vision, were not taking any medications for > 2 weeks, and displayed a normal sinus rhythm on electrocardiogram (ECG) at rest. All subjects gave their written informed consent to participate in this study.

#### Lighting apparatus

**Fig. 1:**
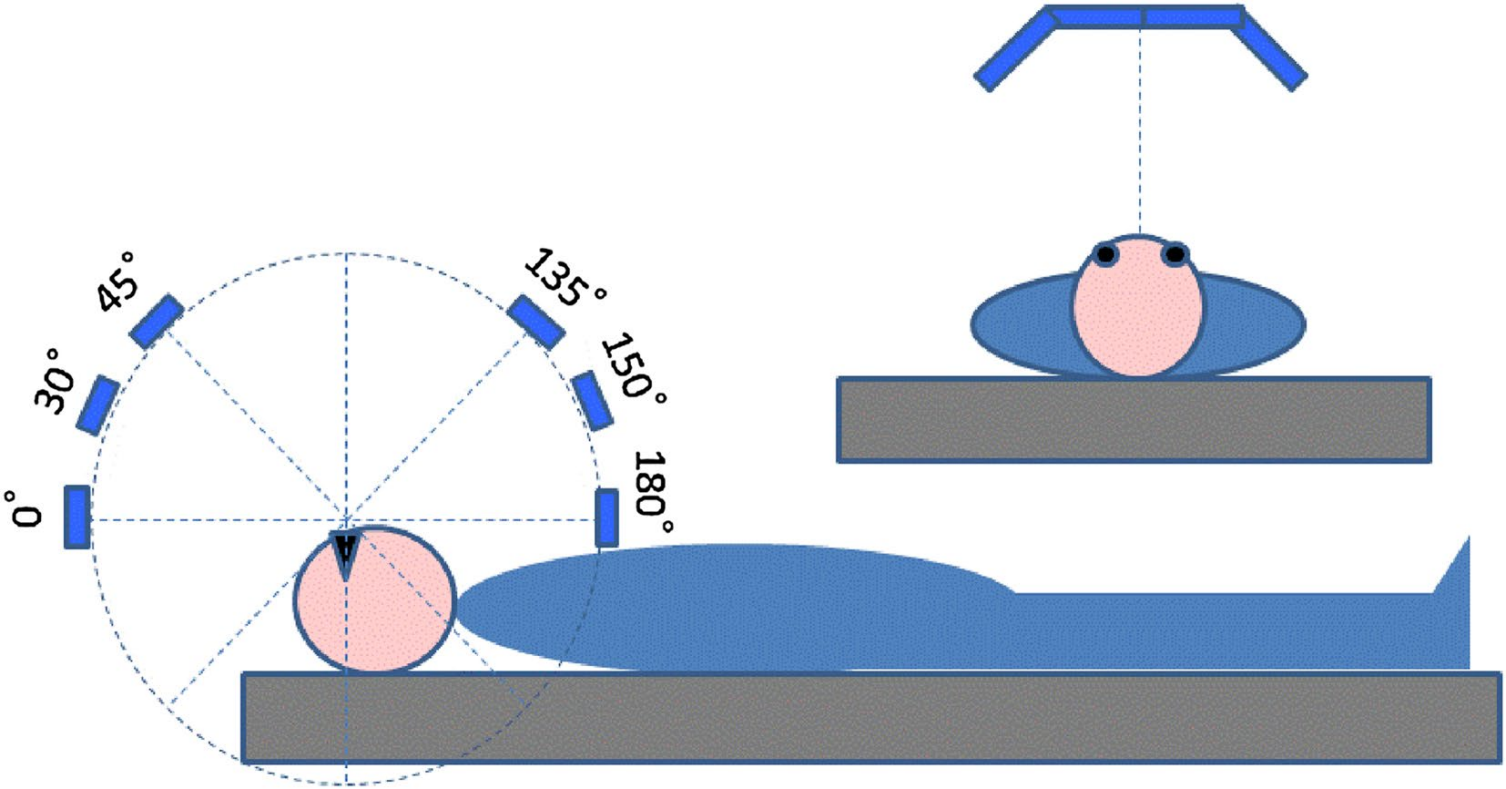
Schema of a lighting device for adjustable illumination angle. The device can be moved in the sagittal plane of subject keeping the distance between the device and subject’s eyes at 24 cm

An organic electroluminescent (OEL) lighting devise developed for research purposes was used. The device consisted of four OEL panels (VELVE OLED Lighting Module with adjustable RGB color and brightness, 55 × 55 mm square, Mitsubishi Chemical Pioneer OLED Lighting Corporation, Tokyo, Japan) that were linearly aligned with two panels at the both sides inclining inward with an angle of 40°. Using a custom-made experimental frame, the lighting device was secured at a constant distance of 24 cm from the eyes of subject lying on a bed in the supine position so that the four OEL panels were aligned across their body axis and able to be moved in their sagittal plane keeping the light axes always facing their eyes (Fig. 1).

Although the device was able to emit red, green, and blue in any combinations, only 100% blue light was used in this study. Front illuminance when illuminated from a 90 degree angle of incidence was 15.4 lx at subject’s eye position, chromaticity (*x*, *y*) was (0.14, 0.16), and the melanopsin-stimulating component that was estimated from the melanoptic spectral efficiency curve adjusted for the effect of human pre-receptoral filtering [17–19] were 75% of the total photon flux density (0.377 µmol/[m^2^ s]). The photo-spectrum of blue light is provided as additional Additional file 1: Figure S1.

#### Study protocol

Subjects were instructed not to consume food or beverages containing caffeine or alcohol after 21:00 the previous night. The experiments were performed between 10:00 and 17:00 in a calm, light-shielded, and air-conditioned (24 ± 2 °C) laboratory more than 2 h after a light meal. Subjects were laid supine on a bed and instructed to continue to look at the mark on the ceiling just above their heads, and not to look directly the light source, while they were exposed to light. The blue light from the device was exposed for 5 min from each of 6 angles (0º, 30º, 45º, 135º, 150º, and 180º). The angle was determined as the angle between the eye and the midpoint of the centerline of the four OLED panels. The order of the angle was counterbalanced among subjects. There was a 10-min dark interval period after light exposure from each angle.

#### Measurement and data analysis

During the experiment including dark and light exposure periods, ECG was recorded continuously with bipolar CM5 lead with a bioelectric amplifier (Biotop mini, East Medic Corporation, Kanazawa, Japan), digitized at 500 Hz with an analog-to-digital converter (AIO-163202FX-USB, CONTEC Corporation, Osaka, Japan), and stored in a hard disk.

From ECG data, R–R interval time series were obtained and analyzed separately for heart rate variability (HRV). The time series data were divided into segments of 5-min light exposure and 10-min dark periods for each incident angle. For each segment, mean heart rate (HR), standard deviation of R–R interval (SDNN), and low-frequency (LF, 0.04–0.15 Hz) and the high-frequency (HF, 0.15-0.40 Hz) components of HRV were computed.

#### Statistical analysis

Statistical analyses system version 9.4 (SAS institute Inc., Cary, NC, USA) was used for the statistical analysis. The Mixed procedure was used for analysis of variance (ANOVA) for repeated measures with incident angle, light-or-dark, and exposure order as the fixed effects and subject as the random effect. According to the guideline of HRV analysis [20], the power of LF and LH component was transformed into logarithmic value to normalize the distribution. P < 0.05 was considered to be statistically significant and Bonferroni adjustment was used to keep type 1 error level in multiple comparisons.

### Results

**Fig. 2:**
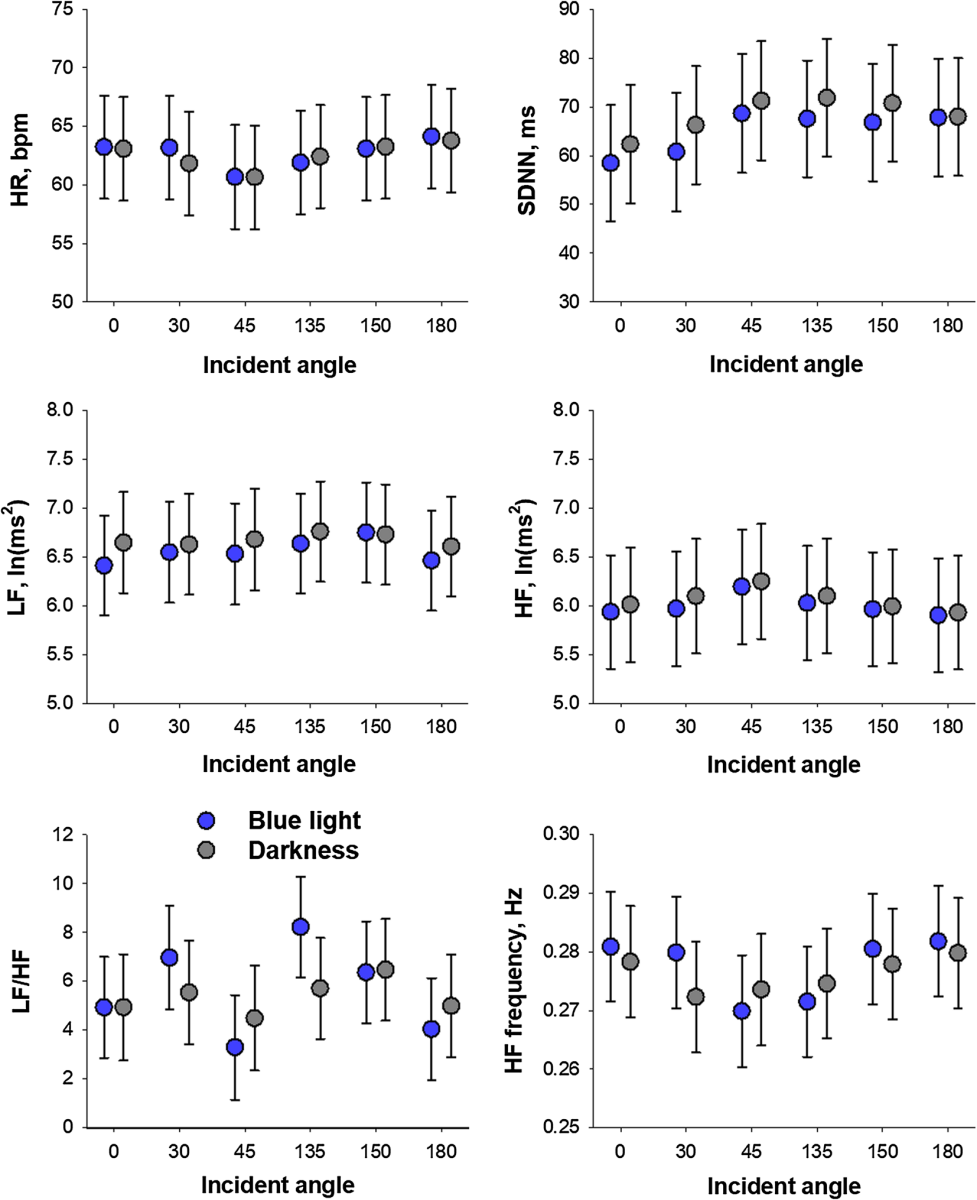
Heart rate (HR) and heart rate variability (HRV) indices during blue light exposure from different incidence angles on the eye (blue bars) and subsequent dark periods (gray bars). Values are least square means and standard errors of the means adjusted for the effects of exposure order. *HF* high frequency, *LF* low frequency, *LF/HF* LF-to-HF power ratio, *SDNN* standard deviation of normal-to-normal R–R intervals

Figure 2 shows HR and HRV indices during blue light exposures and subsequent dark period from different incidence angles. Data are least-square means adjusted for order effect. The statistical analysis for the effects of incident angle, light-or-dark, and order was presented in Table 1.

**Table 1:** Results of repeated ANOVA

	Incident angle		Light vs. dark		Order		Angle × LD^a^		Angle × order^b^	
	*F* (5,70)	*P*	*F* (1,70)	*P*	*F* (1,70)	*P*	*F* (5,70)	*P*	*F* (5,70)	*P*
HR	1.17	0.3	0.17	0.6	2.69	0.1	0.30	0.9	0.69	0.6
SDNN	0.70	0.6	1.27	0.2	15.11	0.0002	0.06	0.9	1.31	0.2
LF power	0.69	0.6	1.12	0.2	12.52	0.0007	0.10	0.9	0.74	0.5
HF power	0.27	0.9	0.69	0.4	11.88	0.001	0.04	0.9	0.73	0.6
LF/HF	0.52	0.7	0.10	0.7	3.14	0.08	0.43	0.8	0.33	0.8
HF frequency	2.16	0.06	0.42	0.5	0.26	0.6	0.70	0.6	1.60	0.1

Abbreviations are explained in legend to Fig. 2

^a^Interaction between incident angle and light vs. dark (LD)

^b^Interaction between incident angle and order

Incident angle showed no significant effect on any autonomic indices of HRV. There was no significant effect of light-vs-dark, interaction between incident angle and light-vs-dark, or interaction between incident angle and incident angle order. On the other hand, significant order effects were observed on SDNN and LF and HF power.

Significant order effects were also detected for HR and HRV indices during dark period (additional Additional file 2: Figure S2). Regardless of the angle of incidence, hear rate and HF frequency decreased by the first two or three light exposures, while SDNN and LF power increased after 4th or 5th exposures.

### Discussion

To examine whether autonomic response to blue light depends on the angle of incidence on the eye, we analyzed HRV during and after blue light exposures from various angles. However, no significant difference was detected in HRV autonomic indices with the angle of incidence.

To our knowledge, this is the first study to analyze the effect of incidence angle on the non-image forming function of blue light. As to white light, however, Lasko et al. [15] reported that nighttime bright white light in the upper visual field caused greater melatonin suppression than the light in the lower visual filed. Similarly, Glickman et al. [16] demonstrated that nighttime white light exposure to the inferior retina caused melatonin suppression, while exposure to the superior retina caused no significant suppression. We have previously observed that exposure to OEL blue light causes suppression of parasympathetic component of HRV, while red and green OEL light show no such effect [21]. These findings suggest that non-image forming effects of light including those on autonomic functions may differ with the incidence angle on the eye. Those motived us to perform the present study, but no clear evidence supporting for the different autonomic effect of blue light with the incidence angle was obtained.

Although the present study failed to detect the significant incidence angle effect on autonomic responses to blue light, it seems important to expand our knowledge of biological effects of blue light. Many authors have warned the health risk of nighttime use of smartphones and tablet devices that emit blue light [8–12]. The displays of these devices use SSLs for the back light of liquid crystal, most of which are white LEDs made from a combination of blue LED at 460 nm and a broad-spectrum yellow garnet phosphor [10, 11]. Even though they appear to emit white light, they emit much blue light that can stimulate melanopsin. Thus, the use of these devices at night is thought to evoke adverse non-image forming responses through ipRGCs. Actually, Chang et al. [13] performed a study with a randomized, cross-over design to compare the effects of reading on an electric book emitting blue light (wavelength, ⁓ 450 nm) and of reading a printed book on sleep. The study consisted of two conditions: (1) 4-h of reading on a light-emitting electric book before bedtime in very dim room light for five consecutive evenings and (2) 4-h reading of a printed book in the same very dim light room before bedtime for five consecutive evening. They observed that, compared with the printed book condition, the electric book condition suppressed evening melatonin levels, delayed dim light melatonin onset, lengthened sleep latency, decreased evening sleepiness, and reduced next morning alertness. Although there are no studies to report the autonomic effect of　the　use　of smartphones or tablet devices, the autonomic effect of blue light itself has been reported previously [21, 22]. In a study of the autonomic neural effects of OEL light with various colors, Yuda et al. [21] demonstrated a greater suppression of cardiac vagal modulation (HF power) by blue light than red and green lights. In this study, the illuminance of blue light was 10 lx at the eyes of subjects lying in the supine position, the light was emitted from OEL panels 24 cm apart from the subjects’ eyes, and the angle of incidence was 90º. To control and reduce the health risk of the use of blue-light-emitting device, further studies are required on the characteristics of the biological effects of blue light.

## Limitations

This study has several limitations. First, the sample size was small and thus, the order effects of incidence angle may not be counterbalanced sufficiently. Second, the amount of time spent gazing at the point on the ceiling during light exposure was not measured. Thus, we cannot exclude the possibility that the difference in the time may have affected on the results. Third, there was only 10-min dark period between the blue light exposures from different angles. This may have been too short to wash out the autonomic effects of preceding incidence angle blue light and may have resulted in an accumulation of the effect. In fact, HR and HRV index during the dark period after light exposure showed significant gradual changes with exposure sequence, regardless of the angle of incidence, suggesting a cumulative effect of time and/or blue light exposures; particularly, deep rest or reduced alertness over time. These effects would overlap with the measurements during light irradiation and may have confounded the effect of incident angle. Fourth, we used OEL as blue light source. Although the spectral distributions of the light showed that the distribution mostly overlaps with the wavelength of melanopsin stimulating component, the results may not represent the features of general SSLs. Fifth, we performed the experiments during daytime. The results may differ with the time of day due to the possible influences of circadian rhythm. Finally, this study was designed to examine the acute autonomic effects of the incidence angle of blue light, but it can affect other non-image forming functions, such as melatonin secretion, circadian phase, sleep quality, and next morning alertness. To determine the long-term effects of incidence angle on these functions, research plans focused on effective irradiation angles are necessary. The present observations are thought to provide useful insights to such future studies.

## Supplementary information

**Additional file 1: Figure S1.** Photo-spectrum of blue light (A) and melanoptic spectral efficiency (B) melanoptic spectral efficiency was calculated from Ref. [17].

**Additional file 2: Figure S2.** HR and HRV indices during baseline dark period and dark period after each light exposure. The data are presented as a function of exposure sequence (order) regardless of the angle of incidence so that the effects of accumulated time and exposed blue light can be accounted for. *Values significantly different (α < 0.05) from baseline control (C) with multiple comparisons. Abbreviations were explained in legend to Fig. 2.

## Data Availability

The datasets used and/or analyzed during the current study are available from the corresponding author on reasonable request.

## Abbreviations

HF: High frequency
HR: Heart rate
HRV: Heart rate variability
ipRGC: Intrinsically photosensitive retinal ganglion cell
LF: Low frequency
LF/HF: LF-to-HF power ratio
OEL: Organic electroluminescent
SDNN: Standard deviation of normal-to-normal R–R intervals
