# Supplementary figures and images for: Difference in autonomic nervous effect of blue light depending on the angle of incidence on the eye
Source: BMC Res Notes. 2020 Mar 10;13:141. doi: 10.1186/s13104-020-04988-5 (PMC7063703; doi:10.1186/s13104-020-04988-5)

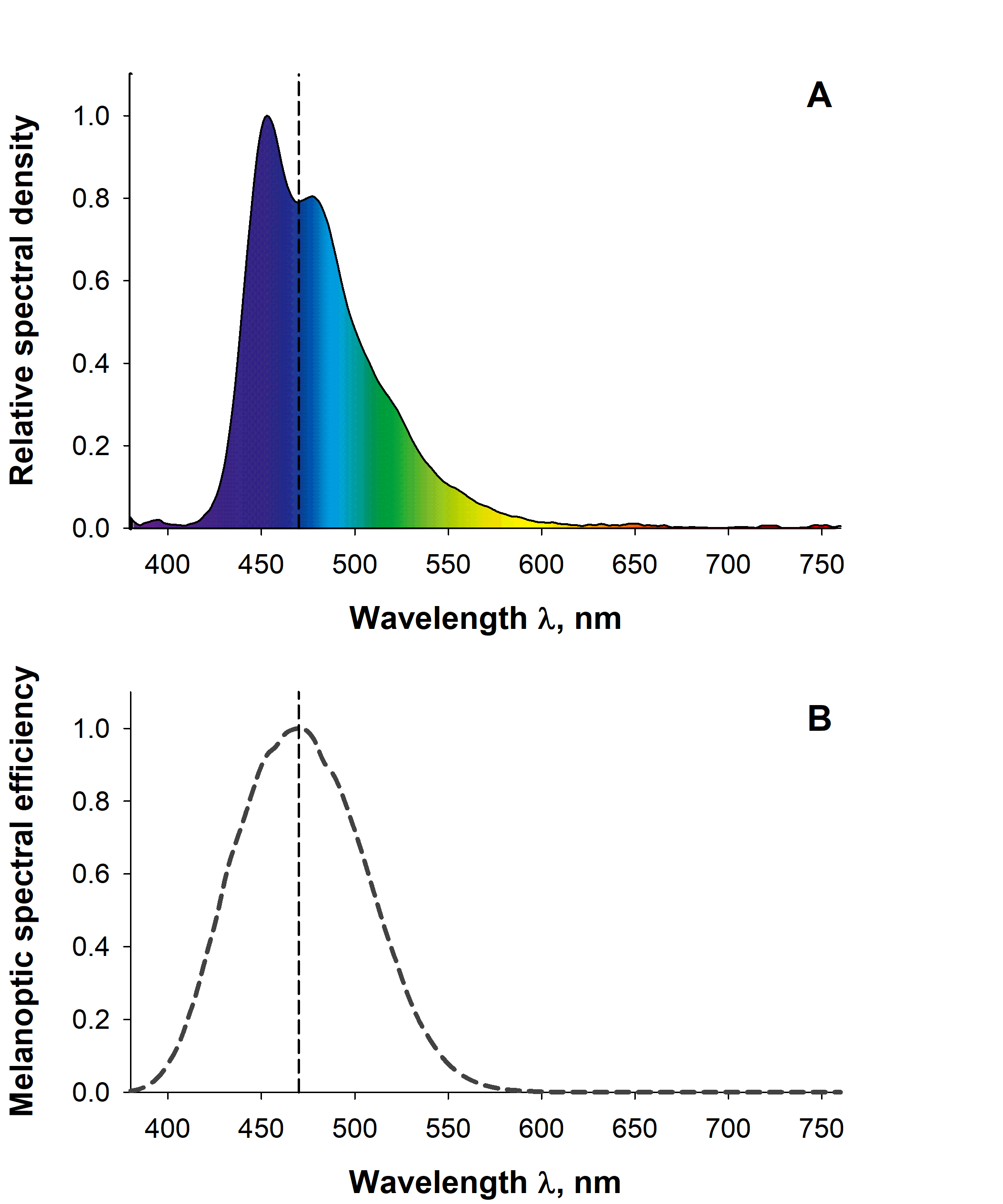

Supplement: Supplementary file 1 — Additional file 1: Figure S1. Photo-spectrum of blue light (A) and melanoptic spectral efficiency (B) melanoptic spectral efficiency was calculated from Ref. [17]. [file 13104_2020_4988_MOESM1_ESM.gif]

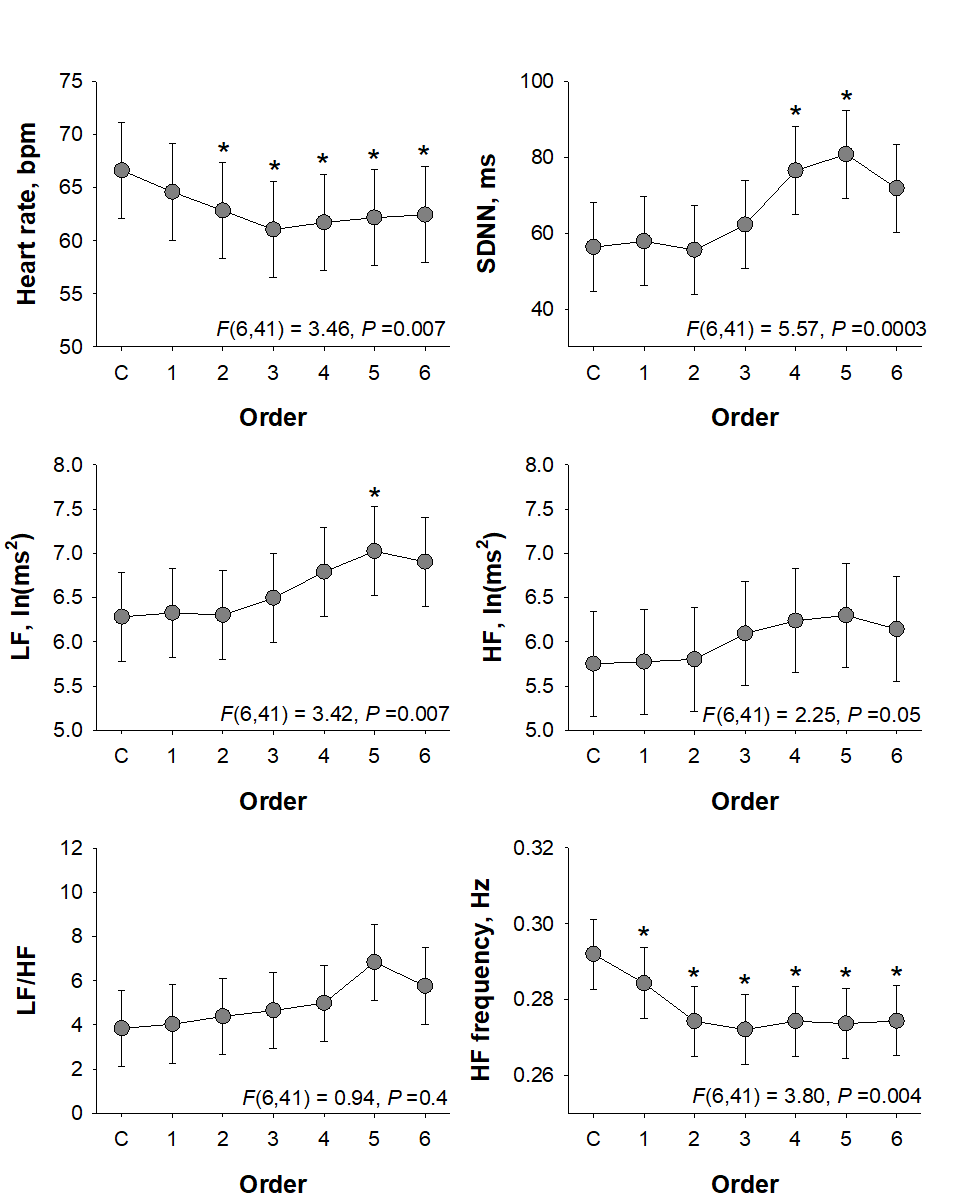

Supplement: Supplementary file 2 — Additional file 2: Figure S2. HR and HRV indices during baseline dark period and dark period after each light exposure. The data are presented as a function of exposure sequence (order) regardless of the angle of incidence so that the effects of accumulated time and exposed blue light can be accounted for. *Values significantly different (α < 0.05) from baseline control (C) with multiple comparisons. Abbreviations were explained in legend to Fig. 2. [file 13104_2020_4988_MOESM2_ESM.gif]
